# Humoral and cellular immune responses to *Yersinia pestis* Pla antigen in humans immunized with live plague vaccine

**DOI:** 10.1371/journal.pntd.0006511

**Published:** 2018-06-11

**Authors:** Valentina A. Feodorova, Anna M. Lyapina, Maria A. Khizhnyakova, Sergey S. Zaitsev, Lidiya V. Sayapina, Tatiana E. Arseneva, Alexey L. Trukhachev, Svetlana A. Lebedeva, Maxim V. Telepnev, Onega V. Ulianova, Elena P. Lyapina, Sergey S. Ulyanov, Vladimir L. Motin

**Affiliations:** 1 Laboratory for Molecular Biology and NanoBiotechnology, Federal Research Center for Virology and Microbiology, Branch in Saratov, Saratov, Russia; 2 Department for Microbiology, Biotechnology and Chemistry, Saratov State Agrarian University named after N.I. Vavilov, Saratov, Russia; 3 Department of Vaccine Control, Scientific Center on Expertise of Medical Application Products, Moscow, Russia; 4 Laboratory of Microbiology of *Yersinia pestis*, Anti-plague Research Institute, Rostov-on-Don, Russia; 5 Department of Pathology, Department of Microbiology & Immunology, University of Texas Medical Branch, Galveston, Texas, United States of America; 6 Department for Infectious Diseases, Saratov State Medical University named after V.I. Razumovsky, Saratov, Russia; 7 Department for Medical Optics, Saratov State University, Saratov, Russia; George Washington University School of Medicine and Health Sciences, UNITED STATES

## Abstract

**Background:**

To establish correlates of human immunity to the live plague vaccine (LPV), we analyzed parameters of cellular and antibody response to the plasminogen activator Pla of *Y*. *pestis*. This outer membrane protease is an essential virulence factor that is steadily expressed by *Y*. *pestis*.

**Methodology/Principal findings:**

PBMCs and sera were obtained from a cohort of naïve (n = 17) and LPV-vaccinated (n = 34) donors. Anti-Pla antibodies of different classes and IgG subclasses were determined by ELISA and immunoblotting. The analysis of antibody response was complicated with a strong reactivity of Pla with normal human sera. The linear Pla B-cell epitopes were mapped using a library of 15-mer overlapping peptides. Twelve peptides that reacted specifically with sera of vaccinated donors were found together with a major cross-reacting peptide IPNISPDSFTVAAST located at the N-terminus. PBMCs were stimulated with recombinant Pla followed by proliferative analysis and cytokine profiling. The T-cell recall response was pronounced in vaccinees less than a year post-immunization, and became Th17-polarized over time after many rounds of vaccination.

**Conclusions/Significance:**

The Pla protein can serve as a biomarker of successful vaccination with LPV. The diagnostic use of Pla will require elimination of cross-reactive parts of the antigen.

## Author summary

*Yersinia pestis*, the causative agent of plague, has been recognized as one of the most devastating pathogen experienced by mankind. It remains endemic in many parts of the world, and is considered emerging pathogen. A live attenuated *Y*. *pestis* strain EV line NIIEG has been used for decades in the former Soviet Union for human vaccination and has proven effective against all forms of plague. We began characterizing the *Y*. *pestis*-specific antibody and T cell-mediated immune responses in people immunized with live plague vaccine. The long term goal of our research is to understand the protective mechanisms underlying immunity to plague in humans and to discover novel protective antigens for their incorporation into a subunit vaccine. Here, we describe our study on immune responses in vaccinees to one of the essential virulence factors of *Y*. *pestis*, namely Pla antigen. The results of the study shed light on the development of the optimal markers to assess the correlation with vaccine-induced protection.

## Introduction

Plague is known as a primary natural zoonosis but is an extremely deadly infection for humans. The disease is caused by *Yersinia pestis*, a gram-negative bacterium, which upon entry in the body of mammalian host is capable of establishing three major forms of plague: bubonic, septicemic, and pneumonic [1, 2]. The plasminogen activator (Pla) of *Y*. *pestis* is an outer membrane protease involved in dissemination of *Y*. *pestis* into circulation, and is one of the major virulence determinants of this pathogen [3–5]. The Pla protein is the surface-exposed trans-membrane β-barrel protease of the Omptin family with homologs found among many bacteria across family Enterobacteriacea [6]. Nevertheless, only Pla can convert plasminogen to plasmin by limited proteolysis, and this activity was likely crucial for the increased lethality of *Y*. *pestis* that developed during the course of evolution [7–9].

Detectable levels of relevant antibodies to Pla (anti-Pla Abs) have been measured in the convalescent sera of human patients who survived plague infection, as well as in mice that survived experimental plague infection [10, 11]. Moreover, anti-Pla Abs of IgG class were detected in the sera of animals and humans vaccinated with live plague vaccine (LPV) indicating immunogenicity of this outer membrane protein [12]. Immunization with purified recombinant Pla or its use in a DNA vaccine formulation provided no protection against plague in a murine model [13]. Nevertheless, partial protection was seen in mice and rats against strain of *Y*. *pestis* lacking capsular antigen F1 [14].

Besides the testing of Pla as a potential protective antigen for plague subunit vaccine formulation, there were attempts to use this outer membrane protein for immuno-diagnostic purposes. A panel of monoclonal antibodies (MAbs) to Pla was created to different epitopes that were either species-specific for *Y*. *pestis* or able to recognize other bacteria [15]. Similar studies resulted in selection of anti-Pla MAbs capable of detecting natural *Y*. *pestis* isolates, as well as modified strains of plague microbe like capsule-negative variants [16, 17].

The live plague vaccine created almost a century ago is still widely used in the former Soviet Union and China to immunize plague researchers and people at risk living in plague endemic territories [12, 18]. The advantage of the LPV over a killed plague vaccine is its ability to defend against all forms of plague, as well its ability to mimic to the plague infectious process to a certain extent, resulting in a robust protection [19]. However, this vaccine is not approved for human use in the Western countries due to the safety concerns [20]. Nevertheless, construction of rationally attenuated vaccine strains of *Y*. *pestis* has garnered attention in recent years [21], especially because the LPVs can induce both humoral and cellular immunity against plague [22–24]. Therefore, a detailed study of human immunity elicited by LPV is beneficial for both understanding the mechanism underlying the immune response to this vaccine and for future evaluation of efficacy of the next generation of plague vaccines.

In this study, we investigated antibody and cell-mediated immunity in individuals vaccinated with the live plague vaccine line EV NIIEG, which is a derivative of the well-known vaccine strain *Y*. *pestis* EV76 [12]. Here, the Pla protein was used as a model antigen, which we intended to utilize in the future as a tool for evaluation of vaccine efficacy of vaccination and as a marker of exposure to plague.

## Methods

### Ethics statement

Each human volunteer provided written informed consent for blood donation. The patients in this manuscript have given written informed consent (as outlined in the PLOS consent form) to publication of their case details. This study was approved by the Human Bioethics Committee of the Saratov Scientific and Research Veterinary Institute. The Institutional Review Board (IRB) was registered with the Office for Human Research Protections (OHRP), registration number IRB00008288 (https://ohrp.cit.nih.gov/search/irbsearch.aspx?styp=bsc).

### Study subjects

Sera from healthy 26–72 years old volunteers (n = 34, group A) of both genders who received multiple annual immunizations (2–51 injections) with the live plague vaccine line EV NIIEG (LPV), as well as from healthy individuals (n = 17, group B) who had no history of contact with either *Y*. *pestis* microbe or its antigens, were tested. We further divided group A of immunized donors into subgroups of recently vaccinated (A-RV, less than one year post-vaccination, n = 13) and early vaccinated (A-EV, more than one year post-vaccination, n = 21). The vaccination was performed by intradermal immunization (scarification), which is a standard way to immunize people with LPV in Russia [12]. This immunization was done to plague researchers in their respectful institutions, and was not performed by us. The sera were aliquoted and stored at -80°C.

### Isolation of PBMCs, proliferation assay, and cytokine profiling

Peripheral blood mononuclear cells (PBMCs) were isolated from heparinized blood by density gradient centrifugation in Histopaque (Sigma, St. Louis, MO) according to standard protocol. Cells were cultured in DMEM/F12 medium containing 10% FBS and antibiotic-antimycotic supplement for six days with or without stimulatory agent in 96 well plates (10^5^ cells per well). The Hig-Tag-labeled *Y*. *pestis* recombinant proteins were purified as described previously for the panel of five antigens [25]. The quality of purification was evaluated with the silver stained PAGE. Soluble antigens, such as F1, were treated with AffiPrep Polymyxin resin (BioRad, Herciles, CA) to remove the traces of LPS, while partially soluble Pla was isolated in two steps. First, we isolated Pla-containing inclusion bodies, and then purified Pla using Ni^2+^-chromatography under denaturing conditions. The level of contaminating LPS was measured with QCL-1000 Chromogenic LAL Assay kit (Fisher Scientific). Both antigens were essentially LPS-free, as the LPS contamination was below the sensitivity level of the kit (0.1–1.0 EU/ml). Unstimulated PBMCs served as negative controls, and Concanavalin A from *Canavalia ensiformis* Type IV-S (ConA) (Sigma) was used as a positive control. The proliferative response was measured in quadruplicate by detection of BrdU incorporation using Cell Proliferation ELISA, BrdU chemiluminescent kit (Roche Applied Science, Indianapolis, IN) according to manufacturer’s protocol. The chemiluminescence was measured by using a BioTek Synergy HT reader (BioTek Instruments Inc., Winooski, VT). The proliferative response was expressed as a stimulation index (SI) calculated by dividing the mean relative light units per second (rlu/s) obtained for the cultured cells with a stimulant by the rlu/s of non-stimulated wells. Culture supernatants were collected on day 5 and preserved at -80°C until further use. The levels of IFN-γ, TNF-α, IL-4, IL-10, and IL-17A were measured by using commercial ELISA kits (Vector-Best, Cytokine, Russia) according to the manufacturer’s instructions. The reaction was developed using streptavidin-horseradish peroxidase with the tetra-methyl benzidine chromogen (TMB), and the optical density was measured at 450 nm.

### Determination of specific IgG antibody titer to Pla by ELISA

Immulon 2 HB plates (Thermo Scientific, USA) were coated overnight at 4°C with recombinant Pla at concentration 5 μg/ml dissolved in 0.1 M carbonate buffer, pH 9.5 with 8 M urea. The remaining binding sites were blocked with 20% Newborn Calf Serum (Sigma) in Phosphate Buffered Saline (PBS). Each serum sample was two-fold serially diluted in the range of 1:50 to 1:800. Goat anti-human IgG (Fab-specific)-peroxidase (HRP) antibody (Sigma) was used as secondary antibody. The reaction was developed with the TMB substrate (Sigma). The bacterial suspension of LPV was used as a control coating antigen in ELISA. The titers were calculated as the last dilution giving values above the cut-off level that was the mean value of the blank wells (sera without antigen).

### Detection of serum immunoglobulin classes and IgG subclasses

Human antibody isotyping was performed by immunoblotting technique using relevant commercial murine monoclonal subtyping IgG subclass antibodies (IgG1, IgG2, IgG3, and IgG4), as well as anti-human IgA, IgM, and IgE class specific antibodies (Rosmedbio Ltd., St.-Petersburg, Russia). The recombinant Pla antigen was separated by 12.5% SDS-PAGE, transferred to a nitrocellulose membrane, incubated with serially diluted human sera, and then probed with corresponding anti-human MAbs. Goat anti-mouse IgG (Fab-specific)-HRP Ab (Sigma) was used as secondary antibody. The substrate was TMB for the membranes (Sigma). The endpoints were determined visually with the signal considered positive when the intensity was twice over the background.

### Linear B-cell epitope mapping

B-cell immune-reactive epitope mapping of the target antigen was performed in ELISA by using a library of 61 peptides generated from the sequence for Pla of *Y*. *pestis* CO92 (accession no. CAB53170.1) and consisting of 15-mer peptides overlapping by 10 amino acids (S1 Table). Nunc Immobilizer, Amino Modules Plates (Thermo Scientific) were coated with 20 μg of individual peptides in 0.1 M carbonate buffer, pH 9.5, overnight. ELISA was then performed as described above. The dilution of tested sera was 1:100. The interpretation of data was performed as described in a previous study with a similar design [26, 27]. Briefly, optical density (OD) values were read with a BioTek Synergy HT reader at a wavelength of 450 nm (reference wavelength, 630 nm). A signal was assigned as positive when it reached the cutoff value of twice the background OD. The background OD was the mean of the lowest 50% of all OD values obtained with that particular serum. The wells containing no peptides were used as negative controls, and recombinant Pla was used as a positive control.

### Statistical analysis

GraphPad Prism 6 software was used for data handling, analysis, and graphic representation. Non-parametric tests, i.e. the Mann–Whitney test for continuous unpaired data and the Chi-square test or the Fisher’s exact test for dichotomous variables, were performed for statistical analysis. Associations were assessed using Spearman’s Rank Correlation coefficient. A P *value* <0.05 was considered statistically significant.

## Results

### Proliferative and cellular response to antigen stimulus

**Fig 1:**
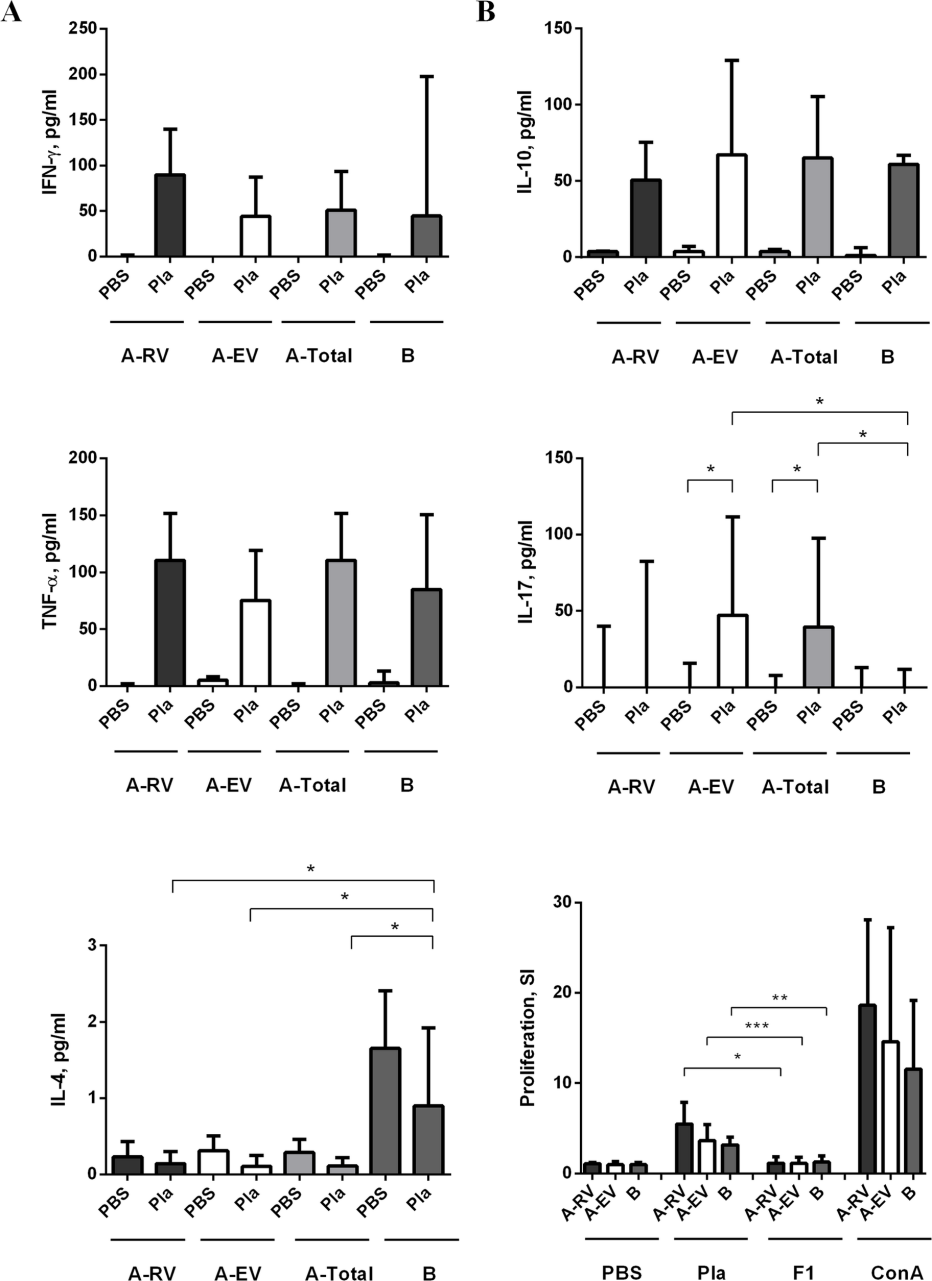
Measurement of cytokines in supernatants of human PBMCs of immunized (group A) and naïve (group B) donors stimulated with recombinant Pla [5 μg/ml] or F1 [2 μg/ml]. Group A was further divided into subgroups A-RV (recently vaccinated, n = 13) and A-EV (earlier vaccinated, n = 21) with post vaccination time less and more than one year, respectively. The SI was calculated against unstimulated cells (PBS). Concanavalin A (Con A) [1 mg/ml] served as positive control stimulus. The concentration of cytokines IL-10, and IL-17A (panel **A**) and IFN-γ, TNF-α, and IL-4 (panel **B**) is given in picograms per milliliter (pg/ml). The bars represent the median ± interquartile range calculated from quadruplicates. The statistical analysis was done by Mann-Whitney test. Statistically significant differences between the groups are indicated by * (*p*<0.05), ** (*p*<0.01), and *** *(p*<0.0001).

To assess the *in vitro* proliferative response, PBMCs isolated from study subjects were stimulated with 5 μg/ml Pla or 2 μg/ml F1 (control antigen). The SI induced by Pla was noticeably higher than that obtained in response to the control F1 antigen in both relevant vaccinated groups, such as group A-RV and group A-EV (*p<*0.05, *p<*0.0001, respectively), as well as in the group B of unvaccinated individuals (*p*<0.01) (Fig 1A). Although the proliferative response to Pla was pronounced, there was no significant difference between both A-RV and A-EV groups of vaccinees and control donors in the group B. Nevertheless, a moderate trend of slightly higher stimulation indexes was observed in the cohort of recently vaccinated individuals (A-RV group) compared with the donors in the A-EV group with the last vaccination occurring more than one year ago (*p =* 0.117).

The *in vitro* proliferative response to Pla was accompanied by a marked but nonspecific (p*>*0.05) release of a number of cytokines, such as IFN-γ, TNF-α, and IL-10 by PBMCs derived from donors of both vaccinated (A-RV and A-EV) and unvaccinated groups (Fig 1). Surprisingly, we found that production of IL-4 was significantly greater in group B than in vaccinated donors. Although there was no significant difference between stimulated and control PBMCs, the level of IL-4 was reduced by 3.4-fold in group A (ARV and A-EV) of vaccinated donors in comparison with group B of naïve donors (Fig 1B). In contrast, PBMCs obtained from donors of the A-EV group, who received multiple immunizations in the past, responded to stimulation with Pla by 14.7-fold increase (*p*<0.05) in making IL-17A over the naïve donors of the group B (Fig 1A, S1 Fig). This remarkable contribution of IL-17A production from the donors of the A-EV group resulted in the overall significant difference between groups A and B vaccinated and naïve donors (*p* = 0.043), while there was no statistical significance for the group A-RV in this category (*p*>0.05). The observed IL-17A release may indicate that the immune response to LPV becomes Th17-polarized over time multiple rounds of vaccination.

There was a significant modest negative correlation between number of immunizations (*r* = -0.475, *p<*0.05) and the IL-4 response in vaccinees, although corresponding correlation with post-vaccination time was negligible (*r* = -0.196, *p*>0.05) (S2 Fig and S3 Fig). Also, there was a slight positive correlation in the levels of IFN-γ (*r* = 0.018, *p* = 0.943), IL-17A (*r* = 0.018, *p* = 0.943) and TNF-α (*r* = 0.229, *p* = 0.361), and negative correlation of IL-10 (*r* = -0.297, *p* = 0.231), with the number of LPV injections. The increase in the level of IFN-γ (*r* = 0.079, *p* = 0.756), IL-17A (*r* = 0.147, *p* = 0.561), and IL-10 (*r* = 0.116, *p* = 0.646) but not TNF-α (*r* = -0.126, *p* = 0.620) may potentially correlate with the post-vaccination time (S3 Fig), although all latter cases were not statistically significant (*p>*0.05). Overall, we found significant association for the IL-4 cytokine, whose levels decreased in donors after an increasing number of vaccinations.

### Antibody response to Pla in sera of donors vaccinated with LPV

**Fig 2:**
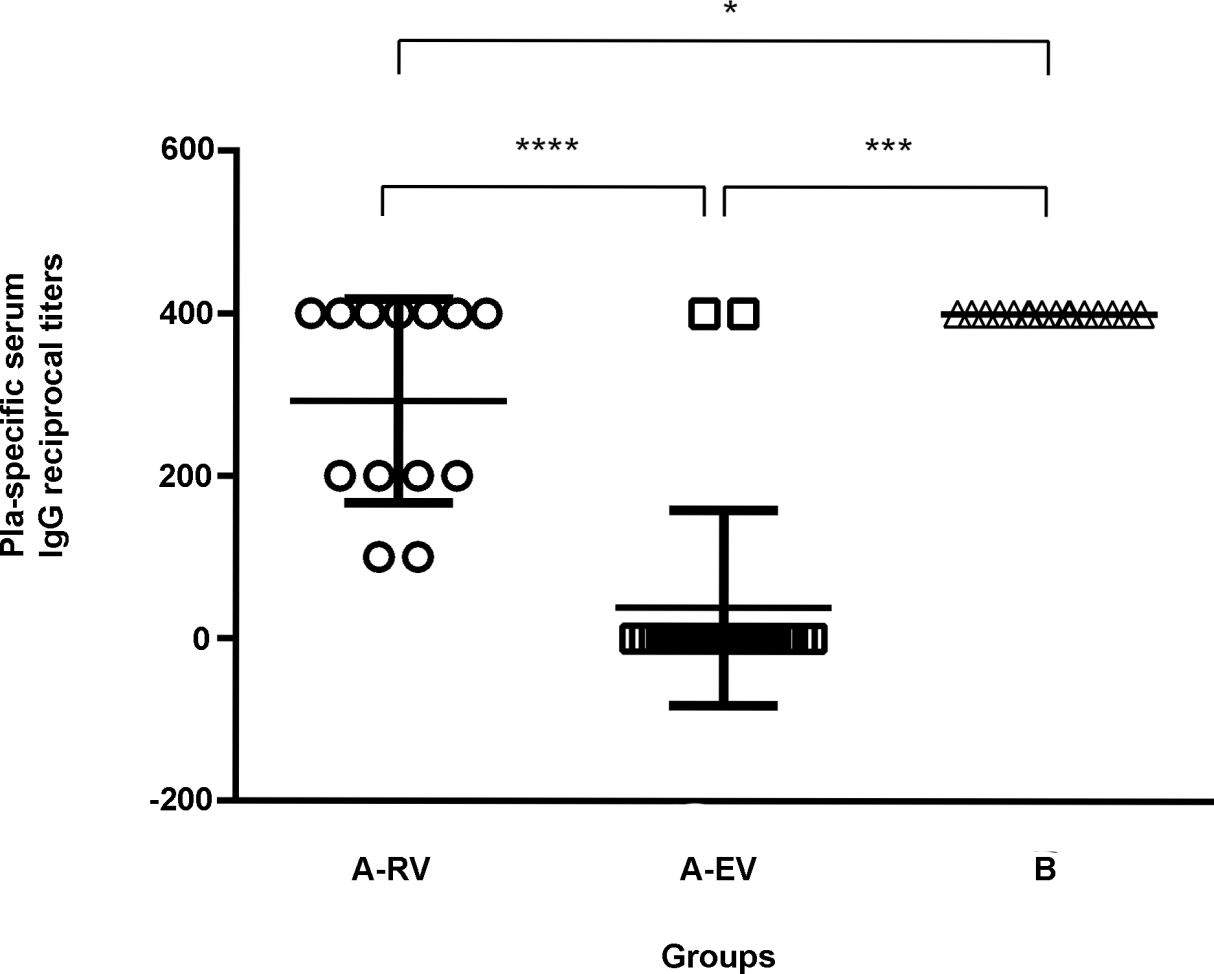
**Individual Pla-specific serum IgG reciprocal titers in groups of recently vaccinated (A-RV), earlier vaccinated (A-EV), and unvaccinated control donors (B).** Data represent antibody titers with standard deviation (SD). The group titer comparative analysis was performed with Mann-Whitney test. Statistically significant differences are indicated by * (*p*<0.05) or *** (*p*<0.0001).

The serological immune response to Pla elicited by the LPV was investigated in the vaccinated donors of group A in comparison with the naïve donors of group B by ELISA. We detected IgG class Abs to Pla, with titers ranging from 1:50 to 1:400, in approximately half of the group A individuals. Moreover, all recently vaccinated donors in the A-RV subgroup were found to be anti-Pla positive. To our surprise, there was a significant difference in both the titers and percent of positive individuals between the subgroups A-RV and A-EV. Donors that received multiple repetitive immunizations (A-EV group) displayed a suppression of the antibody response to Pla. This observation correlates with the negative association between the level of IL-4 and number of LPV immunizations. On the other hand, 100% of the sera collected from donors in the naïve control group B reacted with the Pla antigen and exhibited titers similar to those found in the vaccinated donors of group A-RV, indicating Pla cross-reactivity (Fig 2).

**Fig 3:**
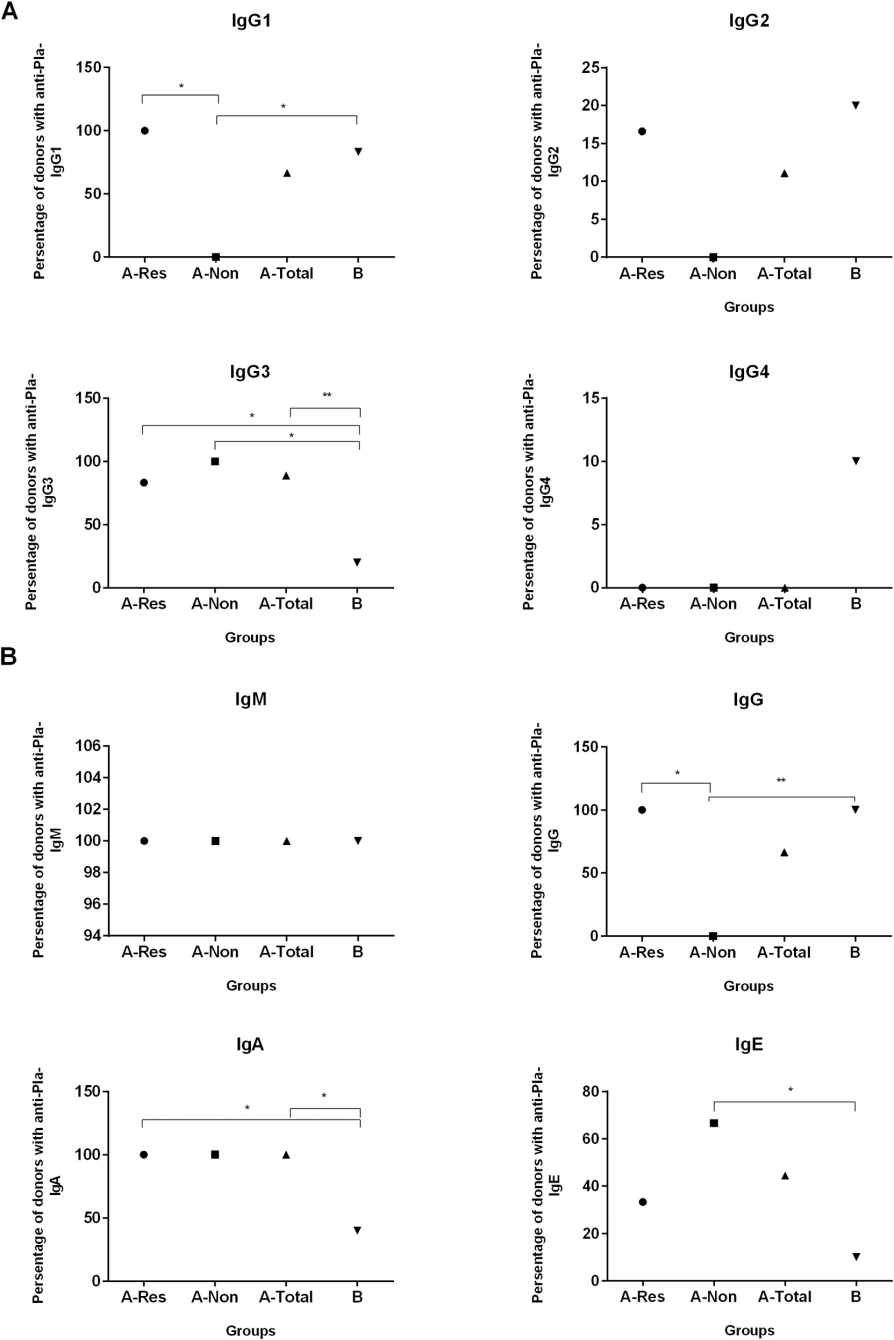
Percent distribution of Pla-specific immunoglobulin classes and IgG subclasses among LPV vaccinated donors which were positive (A-Res) and negative (A-Non) in IgG ELISA with recombinant Pla as coating antigen. A-Total is combined group A, and naïve donors formed the group B. (A) IgG subclasses IgG1, IgG2, IgG3, and IgG4. (B) Antibody classes IgM, IgG, IgA, and IgE. Differences between the groups were statistically compared by the Chi-square test or the Fisher’s exact test when the number of samples was small. Statistically significant differences are indicated by * (*p*<0.05) or by ** (*p*<0.01).

We next determined the reactivity of the sera for anti-Pla antibody classes and IgG subclasses (Fig 3). Based on the ELISA results, we separated the group A donors into responders (A-Res) and non-responders (A-Non). All responders of group A and the majority of positive donors of group B demonstrated immunoreactivity with the IgG1 subclass of immunoglobulins. Only a single individual in each A and B groups showed the reaction with IgG2 subclass (Fig 3A). This one donor from the group A-Res was positive for both IgG1 and IgG2 types. Among donors of the A-Non subgroup the negative reaction was observed for anti-Pla Abs of IgG1 (*p*<0.05), IgG2 and IgG4 subclasses (*p*>0.05). Moreover, all vaccinees (group A) possessed anti-Pla Abs for IgG3 while naïve donors of the group B did not have Pla-specific Abs of this subclass (*p*<0.01). In contrast, anti-Pla Abs of the IgG4 subclass was found exclusively in the sera of the group B donors.

In addition to Pla-specific IgG, we also detected anti-Pla Abs of the IgA class in the sera of vaccinees but not in naïve donors. This corresponded with the increased level of IL-17A released by PBMCs from the group A donors (see Discussion). Also, we observed the presence of anti-Pla Abs of the IgM subclass in both A and B groups of donors (Fig 3B). Finally, IgE class antibodies to Pla antigen were found in sera of about one third of the vaccinated donors, both A-Res and A-Non, and only in a single unvaccinated individual. This result may be indicative of the putative allergenic potential of this antigen and the LPV vaccine in general.

### Mapping immunoreactive linear B-cell epitopes

**Fig 4:**
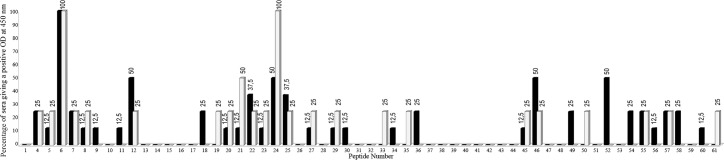
Pla protein-derived overlapping peptides reacting with sera of 8 vaccinated (black columns) and 4 naïve (white columns) donors. A positive result is considered to be at least twice the background OD at 450 nm (background OD range, 0.09 to 0.29). Numbers above columns indicate the actual number of sera reacting with a given peptide.

A library of 61 overlapping peptides, each 15 amino acid residues in length (offset by 5 residues at a time) deduced from the entire Pla sequence was probed with 12 sera samples that exhibited the highest anti-Pla IgG titers determined by ELISA. This set included sera from eight and four donors of the vaccinated A and naïve control B groups, respectively. The results of the screening for each serum are shown in the S2 Table. We found that most of the reactive peptides interacted with Abs from both groups of donors. Nevertheless, the peptides 9, 11, 18, 30, 34, 36, 49, 52, 54, 56, 58, and 60 were specific for the donors of the A group, while peptides 19, 33, 35, 50, and 61 belonged exclusively to the group B donors. The frequency of appearance of each peptide for donor groups A and B is illustrated on Fig 4. Among the group A specific peptides, none reacted with Abs of all eight donors tested indicating the absence of the wide-range immunodominant linear epitope. Only peptide 52 reacted with 50% sera of vaccinated donors, while most of peptides reacted with one or two sera. Therefore, the formation of the antibody response to the Pla antigen with respect to the linear B epitopes might be donor-specific. The B group specific peptides appeared just once in each corresponding serum.

Two peptides, number 6 and 24, were of particular interest, because of their strong cross-reactivity with sera from both vaccinated and naïve donors. Peptide 6 showed the remarkable ability to interact with Abs from any donor of both groups, while peptide 24 reacted 100% with sera from naïve and 50% with sera from vaccinated donors (Fig 4). The existence of two broadly-reactive Pla epitopes may explain the ELISA results shown on Fig 2 in which sera of all naïve donors reacted with the entire Pla antigen immobilized in the wells of the microtiter plate.

## Discussion

In the current study, we investigated for the first time the T-cell recall response to the Pla antigen in human donors vaccinated with LPV. Our data indicate that the proliferative response of human PBMCs to Pla stimulus was strong in nature and even exceeded that induced by capsular F1 antigen, which is known for its pronounced immunogenic characteristics [18, 19]. However, we did not detect a statistical difference in this respect between vaccinated and naïve control donors, suggesting a nonspecific reaction likely due to the presence of cross-reacting T-cell epitope(s) within the Pla antigen. Nevertheless, there was a moderate trend (*p =* 0.117) in observing a slightly high stimulation index in recently vaccinated individuals (less than one year post-immunization). Therefore, we speculate that the specific T-cell response to Pla did occur in this group of vaccinees; however, it was masked by the pronounced cross-reactivity. Also, we report the presence of Pla cross-reactive linear B-cell epitopes that resulted in a strong reaction with this antigen of sera from naïve donors in ELISA. This was not totally surprising to us, since we saw an indication of this antibody cross-reactivity in our previous studies after probing Pla antigen with the panel of monoclonal antibodies [15], and also observed the Pla-reactive band on immunoblot with naïve human sera [25]. Interestingly, multiple vaccinations with LPV suppressed the antibody titers to Pla that were observed when recently (A-RV) and early (A-EV) groups of vaccinated donors were compared (Fig 2). This suppression of the antibody response to the Pla antigen is likely due to the development of a dominant immune response to other competing and more potent antigen(s) of the live *Y*. *pestis* vaccine that became enhanced overtime. If true, this may mean that multiple booster immunizations with LPV may select for the response to a few dominant antigens. These antigens may not even be protective while presenting a threat of developing an allergic reaction instead (see IgE response in Fig 3B).

The Pla protein is considered to be a good candidate for *Y*. *pestis* specific diagnostic antigen [15–17] that is expressed well at both ambient and mammalian host temperatures [28]. However, the observed Pla cross-reactivity may result in certain limitations on its use for diagnostic purposes. Therefore, we mapped the cross-reactive regions of Pla using a library of 15-mer overlapping peptides. Comparison of peptide-ELISA results with sera from eight vaccinated and four naïve donors revealed two major cross-reactive peptides, peptides 6 (IPNISPDSFTVAAST) and 24 (TDHSSHPATNVNHAN). Peptide 6 showed a particularly strong reaction for all sera tested and far exceeded the signal from any other reactive peptide in the library. There were other reacting peptides common for vaccinated and naïve donors; however, they were random and reactive with only one or two sera per group. Importantly, we found 12 peptides that specifically reacted with sera of vaccinated individuals and did not react with sera from naïve donors. Among them, only peptide 52 (TPNAKVFAEFTYSKY) reacted with 50% of sera from vaccinees suggesting that this region can potentially contain an immunodominant linear B-cell epitope recognized by the immune system of humans with different genetic backgrounds. This region may represent a good candidate to test for the purpose of creating a novel plague peptide vaccine.

We determined the distribution of Pla-reacting immunoglobulins within the IgM, IgA, and IgE classes, as well as IgG subclasses (IgG1, IgG2, IgG3, and IgG4) in the sera of vaccinated and naïve donors (Fig 3). The anti-Pla Abs of the IgM class were found in all donors tested. Generally, natural human IgM antibodies or autoantibodies play a role in maintaining the physiological homeostasis and preventing a wide range of different infections [29]. The presence of anti-Pla Abs of IgM class in naïve donors and those who received LPV immunization many years ago suggests that they derived from a constant stimulation of the immune system with cross-reacting antigens rather than from the LPV vaccination. The suspected candidates for these stimulants could be Pla-homologous proteins of the Omptin group found in many Enterobacteriaceae [6]. In contrast, vaccinated, but not naïve, donors contained anti-Pla Abs of IgA class (*p*<0.05) suggesting their origination from LPV immunization by dermal scarification. The existence of Pla-specific IgA correlated with our observation of marked production of IL-17A found after stimulation of PBMCs of vaccinated donors with the Pla antigen (Fig 1A), which was absent in the naïve group of donors. It was shown previously that vaccine-specific Th17 cells formed by parenteral immunization were involved in eliciting a long-term detectible level of secreted IgA [30]. Moreover, subcutaneous priming with recombinant antigen in a Th17-inducing adjuvant followed by boosting promoted high and sustained levels of IgA in the lungs. This response was proven to be associated with germinal center formation in the lung-draining lymph nodes [31]. This may comprehensively explain the high efficiency of LPV against both bubonic and pneumonic plague [12, 18, 24]. Overall, these immune response characteristics to Pla antigen suggest that Th17 polarization of the immunity to LPV can be beneficial to the host during infection [32]. The release of IL-17A in response to stimulation of PBMCs of immunized individuals could also serve as an indicative marker of successful vaccination with LPV. Nevertheless, we would like to speculate that the presence of Pla-specific antibodies of the IgE subclass in vaccinated donors only (Fig 3B) may highlight the danger of a vaccine-related trigger of an allergic response and autoimmune disease. Further studies are needed to shed light on this important issue.

It was reported previously that human immunization with killed plague vaccine induced long-lasting and mixed Th1/Th2 responses that were more polarized towards Th1 [33]. In our study, slightly elevated production of IFN-γ and diminished IL-4 in response to stimulation with Pla in the group of recently vaccinated donors (Fig 1B) also points to a Th1-biased immune response after administration of the live vaccine. This observation is supported by detection of anti-Pla Abs of IgG1 and IgG3, and absence of IgG4 subclasses in the sera of these donors [34–37].

In summary, we found that despite complications with cross-reactivity, human immunity elicited by LPV could be assessed based on analysis of the immune response to Pla antigen. Our analysis showed that LPV vaccination resulted in the response being skewed towards Th1 and Th17, while production of IL-17A by PBMCs of immunized donors in response to Pla antigen stimulation could be a good indicator of the induced immunity. Additionally, we mapped cross-reacting linear B-epitope candidates within the Pla antigen that should be helpful in developing Pla-based diagnostics for *Y*. *pestis*.

## Supporting information

S1 Fig Measurement of IL-17 cytokine in supernatants of human PBMCs of immunized (group A) and naïve (group B) donors stimulated with recombinant Pla [5 µg/ml]. An identical data set was used to draw a corresponding panel on Fig 1, where bars represented the median ± interquartile range calculated from quadruplicates. Here, the same data are shown as mean ± SD. Statistically significant differences between the groups are indicated by * (*p*<0.05).
(DOC)

S2 Fig Analysis of association between cytokine production and a number of vaccinations with the LPV. PBMCs from immunized donors (n = 18) were stimulated with recombinant Pla [5 mg/ml], and supernatants were analyzed for IFN-γ, TNF-α, IL-4, IL-10, and IL-17A levels. The correlation was estimated using Spearman’s Rank Correlation coefficient. The SI was calculated against unstimulated cells. A moderate negative correlation was observed only between IL-4 production versus immunizations received (*r* = -0.4751, *p* = 0.0463).
(DOC)

S3 Fig Analysis of association between cytokine production and post vaccination time in years. PBMCs from immunized donors (n = 18) were stimulated with recombinant Pla [5 mg/ml] and supernatants were analyzed for IFN-γ, TNF-α, IL-4, IL-10, and IL-17A levels. The correlation was calculated with Spearman’s Rank Correlation coefficient. No correlation was revealed for all tested cytokines, as well as between proliferation (SI) and post-immunization time (*p*>0.05).
(DOC)

S1 Table Library of 61 overlapping 15-mer synthetic peptides designed based on 312 amino acid sequence of the Pla protein from *Y*. *pestis* CO92 (GenBank accession no. CAB53170.1). The peptides were made with >95% purity (GenScript, Piscataway, NJ) and stored in aliquots (stock concentration of 10 mg/ml) at -80°C.
(DOC)

S2 Table Immuno-reactive peptides revealed by library screening with sera of vaccinated and naïve donors. (DOC)
