# Supplementary material for: Humoral and cellular immune responses to Yersinia pestis Pla antigen in humans immunized with live plague vaccine
Source: PLoS Negl Trop Dis. 2018 Jun 11;12(6):e0006511. doi: 10.1371/journal.pntd.0006511 (PMC5995359; doi:10.1371/journal.pntd.0006511)
Supplement: S1 Fig — An identical data set was used to draw a corresponding panel on Fig 1, where bars represented the median ± interquartile range calculated from quadruplicates. Here, the same data are shown as mean ± SD. Statistically significant differences between the groups are indicated by * (p<0.05). (DOC) [file pntd.0006511.s001.doc]

**
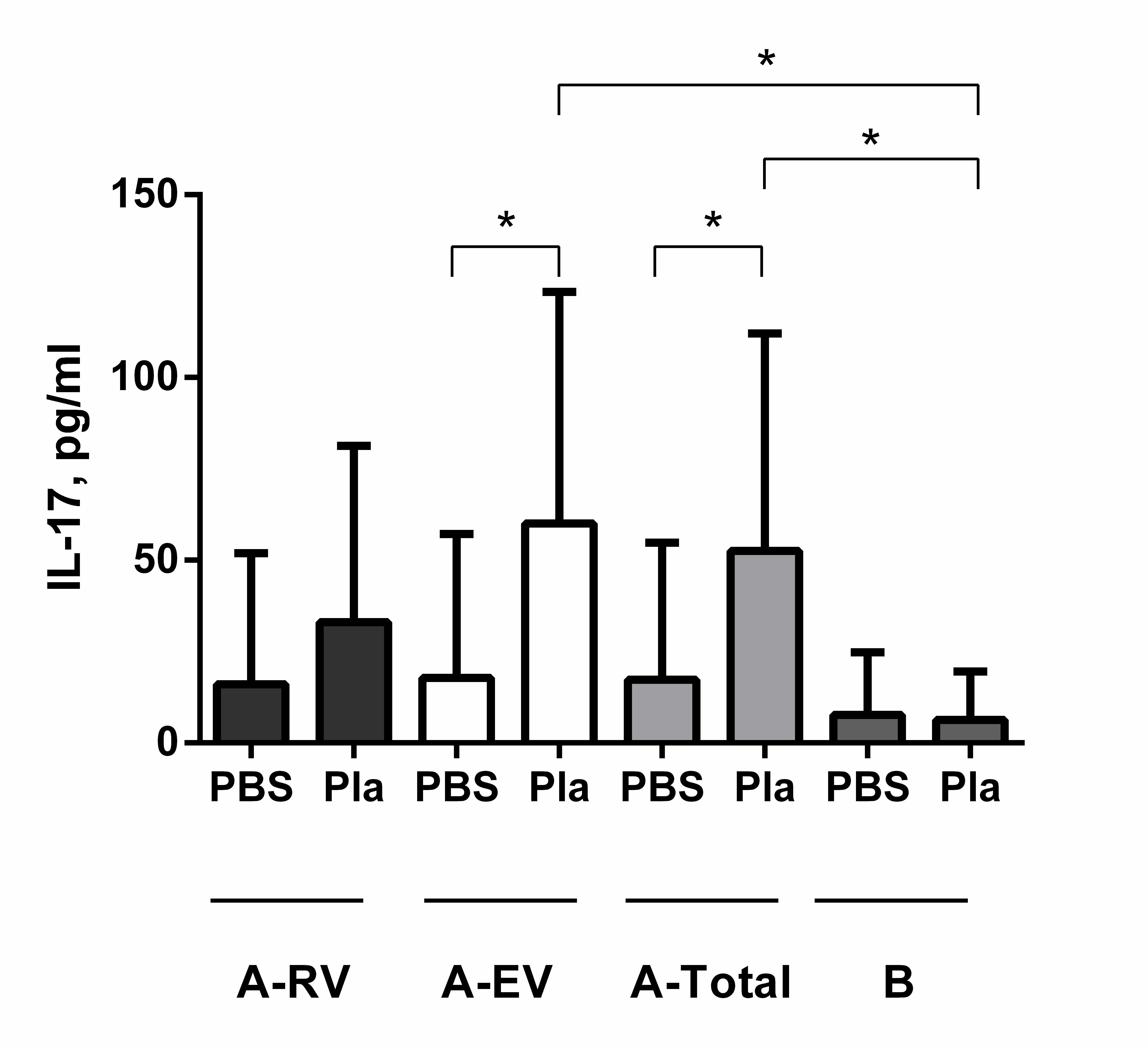
**

**S1 Fig.** Measurement of IL-17 cytokine in supernatants of human PBMCs of immunized (group A) and naïve (group B) donors stimulated with recombinant Pla [5 µg/ml]. An identical data set was used to draw a corresponding panel on Fig 1, where bars represented the median + interquartile range calculated from quadruplicates. Here, the same data are shown as mean + SD. Statistically significant differences between the groups are indicated by * (*p*<0.05).
