# Supplementary material for: Humoral and cellular immune responses to Yersinia pestis Pla antigen in humans immunized with live plague vaccine
Source: PLoS Negl Trop Dis. 2018 Jun 11;12(6):e0006511. doi: 10.1371/journal.pntd.0006511 (PMC5995359; doi:10.1371/journal.pntd.0006511)
Supplement: S2 Fig — PBMCs from immunized donors (n = 18) were stimulated with recombinant Pla [5 mg/ml], and supernatants were analyzed for IFN-γ, TNF-α, IL-4, IL-10, and IL-17A levels. The correlation was estimated using Spearman’s Rank Correlation coefficient. The SI was calculated against unstimulated cells. A moderate negative correlation was observed only between IL-4 production versus immunizations received (r = -0.4751, p = 0.0463). (DOC) [file pntd.0006511.s002.doc]

**S1 Fig.** Analysis of association between cytokine production and a number of vaccinations with the LPV. PBMCs from immunized donors (n=18) were stimulated with recombinant Pla [5 mg/ml], and supernatants were analyzed for IFN-γ, TNF-α, IL-4, IL-10, and IL-17A levels. The correlation was estimated using Spearman’s Rank Correlation coefficient. The SI was calculated against unstimulated cells. A moderate negative correlation was observed only between IL-4 production versus immunizations received (*r* = -0.4751, *p* = 0.0463).
