# Supplementary material for: Humoral and cellular immune responses to Yersinia pestis Pla antigen in humans immunized with live plague vaccine
Source: PLoS Negl Trop Dis. 2018 Jun 11;12(6):e0006511. doi: 10.1371/journal.pntd.0006511 (PMC5995359; doi:10.1371/journal.pntd.0006511)
Supplement: S3 Fig — PBMCs from immunized donors (n = 18) were stimulated with recombinant Pla [5 mg/ml] and supernatants were analyzed for IFN-γ, TNF-α, IL-4, IL-10, and IL-17A levels. The correlation was calculated with Spearman’s Rank Correlation coefficient. No correlation was revealed for all tested cytokines, as well as between proliferation (SI) and post-immunization time (p>0.05). (DOC) [file pntd.0006511.s003.doc]

**S2 Fig.** Analysis of association between cytokine production and post vaccination time in years. PBMCs from immunized donors (n=18) were stimulated with recombinant Pla [5 mg/ml] and supernatants were analyzed for IFN-γ, TNF-α, IL-4, IL-10, and IL-17A levels. The correlation was calculated with Spearman’s Rank Correlation coefficient. No correlation was revealed for all tested cytokines, as well as between proliferation (SI) and post-immunization time (*p*>0.05).
