# Supplementary material for: Humoral and cellular immune responses to Yersinia pestis Pla antigen in humans immunized with live plague vaccine
Source: PLoS Negl Trop Dis. 2018 Jun 11;12(6):e0006511. doi: 10.1371/journal.pntd.0006511 (PMC5995359; doi:10.1371/journal.pntd.0006511)
Supplement: S1 Table — The peptides were made with >95% purity (GenScript, Piscataway, NJ) and stored in aliquots (stock concentration of 10 mg/ml) at -80°C. (DOC) [file pntd.0006511.s004.doc]

**S1 Table.** Library of 61 overlapping 15-mer synthetic peptides designed based on 312 amino acid sequence of the Pla protein from *Y. pestis* CO92 (GenBank accession no. CAB53170.1). The peptides were made with >95% purity (GenScript, Piscataway, NJ) and stored in aliquots (stock concentration of 10 mg/ml) at -80°C.

| Peptide ID | Peptide  length | Sequence |
| --- | --- | --- |
|
| 1. Pla_1-15 | 15 | MKKSSIVATIITILS |
| 2. Pla_6-20* | 15 | IVATIITILSGSANA |
| 3. Pla_11-25* | 15 | ITILSGSANAASSQL |
| 4. Pla_16-30 | 15 | GSANAASSQLIPNIS |
| 5. Pla_21-35 | 15 | ASSQLIPNISPDSFT |
| 6. Pla_26-40 | 15 | IPNISPDSFTVAAST |
| 7. Pla_31-45 | 15 | PDSFTVAASTGMLSG |
| 8. Pla_36-50 | 15 | VAASTGMLSGKSHEM |
| 9. Pla_41-55 | 15 | GMLSGKSHEMLYDAE |
| 10. Pla_46-60 | 15 | KSHEMLYDAETGRKI |
| 11. Pla_51-65 | 15 | LYDAETGRKISQLDW |
| 12. Pla_56-70 | 15 | TGRKISQLDWKIKNV |
| 13. Pla_61-75 | 15 | SQLDWKIKNVAILKG |
| 14. Pla_66-80 | 15 | KIKNVAILKGDISWD |
| 15. Pla_71-85 | 15 | AILKGDISWDPYSFL |
| 16. Pla_76-90 | 15 | DISWDPYSFLTLNAR |
| 17. Pla_81-95 | 15 | PYSFLTLNARGWTSL |
| 18. Pla_86-100 | 15 | TLNARGWTSLASGSG |
| 19. Pla_91-105 | 15 | GWTSLASGSGNMDDY |
| 20. Pla_96-110 | 15 | ASGSGNMDDYDWMNE |
| 21. Pla_101-115 | 15 | NMDDYDWMNENQSEW |
| 22. Pla_106-120 | 15 | DWMNENQSEWTDHSS |
| 23. Pla_111-125 | 15 | NQSEWTDHSSHPATN |
| 24. Pla_116-130 | 15 | TDHSSHPATNVNHAN |
| 25. Pla_121-135 | 15 | HPATNVNHANEYDLN |
| 26. Pla_126-140 | 15 | VNHANEYDLNVKGWL |
| 27. Pla_131-145 | 15 | EYDLNVKGWLLQDEN |
| 28. Pla_136-150 | 15 | VKGWLLQDENYKAGI |
| 29. Pla_141-155 | 15 | LQDENYKAGITAGYQ |
| 30. Pla_146-160 | 15 | YKAGITAGYQETRFS |
| 31. Pla_151-165 | 15 | TAGYQETRFSWTATG |
| 32. Pla_156-170 | 15 | ETRFSWTATGGSYSY |
| 33. Pla_161-175 | 15 | WTATGGSYSYNNGAY |
| 34. Pla_166-180 | 15 | GSYSYNNGAYTGNFP |
| 35. Pla_171-185 | 15 | NNGAYTGNFPKGVRV |
| 36. Pla_176-190 | 15 | TGNFPKGVRVIGYNQ |
| 37. Pla_181-195 | 15 | KGVRVIGYNQRFSMP |
| 38. Pla_186-200 | 15 | IGYNQRFSMPYIGLA |
| 39. Pla_191-205 | 15 | RFSMPYIGLAGQYRI |
| 40. Pla_196-210 | 15 | YIGLAGQYRINDFEL |
| 41. Pla_201-215 | 15 | GQYRINDFELNALFK |
| 42. Pla_206-220 | 15 | NDFELNALFKFSDWV |
| 43. Pla_211-225 | 15 | NALFKFSDWVRAHDN |
| 44. Pla_216-230 | 15 | FSDWVRAHDNDEHYM |
| 45. Pla_221-235 | 15 | RAHDNDEHYMRDLTF |
| 46. Pla_226-240 | 15 | DEHYMRDLTFREKTS |
| 47. Pla_231-245 | 15 | RDLTFREKTSGSRYY |
| 48. Pla_236-250 | 15 | REKTSGSRYYGTVIN |
| 49. Pla_241-255 | 15 | GSRYYGTVINAGYYV |
| 50. Pla_246-260 | 15 | GTVINAGYYVTPNAK |
| 51. Pla_251-265 | 15 | AGYYVTPNAKVFAEF |
| 52. Pla_256-270 | 15 | TPNAKVFAEFTYSKY |
| 53. Pla_261-275 | 15 | VFAEFTYSKYDEGKG |
| 54. Pla_266-280 | 15 | TYSKYDEGKGGTQTI |
| 55. Pla_271-285 | 15 | DEGKGGTQTIDKNSG |
| 56. Pla_276-290 | 15 | GTQTIDKNSGDSVSI |
| 57. Pla_281-295 | 15 | DKNSGDSVSIGGDAA |
| 58. Pla_286-300 | 15 | DSVSIGGDAAGISNK |
| 59. Pla_291-305 | 15 | GGDAAGISNKNYTVT |
| 60. Pla_296-310 | 15 | GISNKNYTVTAGLQY |
| 61. Pla_301-312 | 12 | NYTVTAGLQYRF |

*Hydrophobic peptides, synthesis problem
