# Supplementary material for: Humoral and cellular immune responses to Yersinia pestis Pla antigen in humans immunized with live plague vaccine
Source: PLoS Negl Trop Dis. 2018 Jun 11;12(6):e0006511. doi: 10.1371/journal.pntd.0006511 (PMC5995359; doi:10.1371/journal.pntd.0006511)
Supplement: S2 Table — (DOC) [file pntd.0006511.s005.doc]

**S2 Table. Immuno-reactive peptides revealed by library screening with sera of vaccinated and naïve donors**

| Cohort | Donor # | Peptide ID with positive reaction* | Number of immuno-reactive peptides |
| --- | --- | --- | --- |
| Group A, vaccinated | A11 | 6, 12, **18**, 22, 24, 25, **36**, 46, **54**, **56**, 57, **58** | 12 |
|  | A12 | 6, 12, 46, **52**, 55 | 5 |
|  | A14 | 6, 12, **49, 52** | 4 |
|  | A16 | 4, 6, 7, **18**, 22, 24, 25, **30**, 46, **54, 60** | 11 |
|  | A21 | 6, **49, 52** | 3 |
|  | A25 | 4, 5, 6, 7, 8, **9, 11**, 12, 20, 21, 22, 23, 24, 25, 27, 29, **36**, 45, 55 | 19 |
|  | A27 | 6, 24, 57, **58** | 4 |
|  | A28 | 6, **34**, 46, **52** | 4 |
| Group B, naive | B15 | 4, 5, 6, 7, 8, 20, 21, 22, 23, 24, 25, 27, 29, 33, 45, 50, 55 | 17 |
|  | B16 | 6, 24 | 2 |
|  | B17 | 6, 12, 19, 21, 24, 35, 46 | 7 |
|  | B25 | 6, 24, 25, 46, 57, 61 | 6 |

* Peptides reactive only within the either group A or B are in bold or underlined, respectively. Other peptides are common for both groups.
